# Immune Mechanisms of Filamentous Fungal Keratitis

**DOI:** 10.7759/cureus.61954

**Published:** 2024-06-08

**Authors:** Alexandra Mpakosi, Christiana Kaliouli-Antonopoulou

**Affiliations:** 1 Department of Microbiology, General Hospital of Nikaia "Agios Panteleimon", Piraeus, GRC; 2 Department of Immunology, General Hospital of Nikaia "Agios Panteleimon", Piraeus, GRC

**Keywords:** immune response, aspergillus spp, fusarium spp, infectious keratitis, filamentous fungi

## Abstract

Filamentous fungal keratitis is a particularly serious eye infection that often results in ulceration, corneal perforation, and blindness. The cornea acts as a natural barrier against harmful agents due to the close connection of its epithelial cells. In addition, on its surface, there is a large number of substances with anti-inflammatory and bactericidal properties, such as secretory IgA and mucin glycoproteins, and antimicrobial peptides (AMPs), such as human β-defensin 2 (HBD-2) and LL-37, which are especially increased in filamentous fungal keratitis.

The interaction between pathogenic fungi and the host's immune mechanisms is a complex process: pathogen-associated molecular pattern (PAMP) molecules (chitin, β-glucan, and mannan) found in the fungal cell wall are recognized by pattern recognition receptors (PRRs) (toll-like receptors {TLRs}, C-type lectin receptors {CLRs}, nucleotide-binding oligomerization domain-like receptors {NLRs}, and scavenger receptors {SR}) found in host defense cells, triggering the secretion of various types of cytokines, such as interleukins (IL), tumor necrosis factors (TNFs), and chemokines, which recruit macrophages and neutrophils to migrate to the site of infection and activate inflammatory responses.

In addition, the interaction of hyphae and corneal epithelial cells can activate cluster of differentiation (CD) 4+ T cells, CD8+ T cells, and B cells and induce secretion of T-helper (Th)-type cytokines 2 (IL-4 and IL-13) and IgG.

## Introduction and background

Infectious keratitis is a serious disease that leads to more than 1.5-2 million new cases of complete vision loss or unilateral blindness each year [1]. Bacterial keratitis appears to predominate in different countries and continents, including the United Kingdom, North and South America, the Middle East, and Australia [1].

Fungal keratitis is an urgent, serious infection, more common in tropical developing countries, where it may account for up to 67% of all cases of infectious keratitis [2]. In these climates, filamentous fungi have been found to be the predominant causative organisms, with injury being the most common risk factor.

In contrast, in developed countries, particularly in Europe and the USA, limited cases of fungal keratitis are reported. In these areas, it is considered a rare, often undiagnosed eye infection, most commonly associated with contact lens wear [3-5].

Filamentous fungal keratitis presents challenges in both diagnosis and treatment, has a worse outcome than bacterial keratitis, and is globally considered one of the main causes of vision reduction and/or blindness [5]. Therefore, this infection should be considered an urgent need that requires the increased awareness of the ophthalmologist for a correct clinical diagnosis and immediate treatment. However, very often, even after early treatment, vision loss is inevitable. In addition, antifungal agents have limited activity and ability to penetrate deeper layers of the stratum corneum exhibiting reduced efficacy and increased toxicity. The present study attempts to shed light on the immune mechanisms related to this serious infection, contributing to its understanding and better treatment.

## Review

Epidemiology of infectious keratitis

Zhang et al. recently presented a 20-year review on bacterial keratitis including 21 countries (35 cities). According to their study, the percentage of positive cultures was 47%, while the main causative factors were Gram-positive cocci (62%), Gram-negative bacteria (30%), Gram-positive bacilli (5%), and Gram-negative cocci (5%). The most frequent bacterial species isolated were *Staphylococcus* spp. (41.4%), *Pseudomonas* spp. (17.0%), *Streptococcus* spp. (13.1%), *Corynebacterium* sp. (6.6%), and *Moraxella* spp. (4.1%) [1].

Acharya et al. also studied 625 keratitis cases of which 393 (62.9%) had positive cultures. Bacteria were isolated in 238/393 (60.6%) cases and fungi in 143/393 (36.4%). Gram-positive cocci predominated: *Staphylococcus* spp. (43.7%) and *Streptococcus* spp. (16.4%). Among the Gram-negative bacteria, multiresistant *Pseudomonas* spp. prevailed (13.4%). In 151/625 cases (24.2%), an ocular trauma was mentioned as the main risk factor, followed by previous operations (17.8%), the use of corticosteroids (15.5%), and diabetes mellitus (eight, 3%); 64/151 (42.4%) of the injuries were caused by plant material (48 males versus 16 females). Fungi were isolated in 22 of these injury cases, mainly *Fusarium* spp. (37.1%), *Aspergillus* spp. (30%), and unidentified Phaeohyphomycetes (17.5%); bacteria in 15; both fungi and bacteria in one; and no microorganism in 26 [6].

In 2020, Brown et al. [3] first estimated the global annual incidence of fungal keratitis to be 1,051,787 cases. They assumed that if all the undiagnosed cases of microbial keratitis with false-negative cultures, which are actually of fungal etiology, were added to the above, the global incidence would amount to 1,480,916 cases/year, with the highest values in Asia and Africa and the lowest in Europe. They also estimated that nearly 10%-25% of the eyes with fungal keratitis would eventually perforate or require extraction, while at least 60% of patients (800,000 people/year) would remain monocular even if treated. Brown et al. estimated that 84,143-115,697 eyes are surgically removed worldwide each year and that especially in low- and middle-income countries, 610,821 eyes are blinded annually due to fungal keratitis [3].

More recently, in 2021, Hoffman et al. further estimated that 1.2%-14.0% of all cases of microbial keratitis in Europe and North America were of fungal etiology, with the respective rates being 37.7%-81.5% in tropical and subtropical sub-Saharan Africa and South Asia. In these countries, high levels of humidity and heavy rainfall favor the growth of fungi. In addition, these are developing countries, based on an agricultural economy, with rural workers that are therefore exposed to eye injuries from plant-derived materials, with subsequent damage to the epithelial barrier and fungal invasion of the cornea [7]. Also, many of these patients living in remote rural areas neglect going, do not present, or delay in presenting themselves to the ophthalmologist, due to the long distance, cost of treatment, and loss of daily wages or the lack of an attendant. This delay leads to a poor outcome as by the time they decide to seek medical help, the inflammation has often progressed and the fungus has already penetrated the deeper layers of the eye where topical antifungals are not effective [8,9].

Hoffman et al. also observed that the incidence of fungal keratitis varied with time. This change was more pronounced in developing countries such as Thailand, where the average incidence was 13.6% between 1982 and 2003, and it rose to 50.8% between 2003 and 2006. The same happened in Nepal where the rate increased from 23.1% in 1981 to 70% in 2011, as well as in Ghana (Africa), from 56.1% in 1995 to 74.7% between 1999 and 2001. The possible causes of these changes included climate warming with increased humidity, the widespread use of topical antibiotics and/or topical corticosteroids as initial empiric treatment, the more frequent appearance of risk factors such as diabetes mellitus, the increase in the use of contact lenses, and the improvement of laboratory methods that led more often both in correct diagnosis and in better surveillance and reporting of cases [7].

The incidence of fungal keratitis varies not only between states but also between different regions of each country, mainly due to different risk factors, the mode of infection, and the species of fungus. Therefore, the knowledge of local epidemiology is crucial for choosing the most appropriate treatment. In addition, surveillance can detect emerging fungal species and local outbreaks in time [8].

Recently, there has been a dramatic increase in filamentous fungal keratitis cases worldwide. As mentioned above, most cases occur in developing countries with a tropical/subtropical climate and are usually caused after corneal traumatism [8]. *Fusarium* spp., *Aspergillus* spp., and Phaeohyphomycetes predominate as etiological factors [9]. Moreover, in developed countries, there is an increase in *Fusarium* keratitis associated with contact lens wear [7].

In a Panhellenic multicenter, prospective 16-year study, the first in Greece, a total of 35 cases of filamentous fungal keratitis were identified. The male/female ratio was 1.7:1, and the median age was 48 years. Corneal injury from plant material and soft contact lens wear were the main risk factors (42.8% and 31.4%, respectively). Among the causative agents, *Fusarium* species were most frequently isolated (n=21, 61.8%). *Fusarium solani* was mainly associated with trauma while *F. verticillioides* and *F. proliferatum* with soft contact lens wear. Other fungi that were isolated were the following: *Purpureocillium lilacinum* (14.7%), *Alternaria* spp. (11.8%), *Aspergillus* spp. (8.8%), *Phoma foliaceiphila* (one case), *Beauveria bassiana* (one case), and *Curvularia spicifera* (one case) [10].

Eye anatomy

The sensory organ of vision is the eye. The eye consists of the orbital cavity, the lacrimal drainage system, the eyelids, and the main part of the eye, the eyeball, which is located inside the orbital cavity. The parts that are of particular importance in the study of ocular infections are the cornea, choroid (choroid, iris, and ciliary body), retina, conjunctiva, sclera, vitreous, and aqueous liquid [11].

The eyelids are muscular folds of skin, which protect the eye from various harmful effects of the external environment, and ensure constant moisture in the cornea. In addition, the free lip presents the eyelashes, the sebaceous glands of Zeiss, and the sweat glands of Moll. The lacrimal apparatus produces tears (via the secretory gland) and drains them into the nasal cavity (via the draining gland). Disorders in their secretion or composition cause damage to the conjunctiva and cornea [12,13].

The conjunctiva is a thin mucous membrane that covers the back surface of the eyelids. It consists of two layers: the stratified columnar epithelium and the underlying dermis. The conjunctival epithelium of the sclero-corneal border is of great physiological importance because it is the source of the production of new corneal epithelial cells in cases of total apoptosis of the corneal epithelium.

The cornea is the main refractive lining of the eye. It is a vascular tissue that occupies the anterior 1/6 of the eyeball. It is transparent and consists of five layers [14]: the stratified epithelium of the cornea; Bowman's membrane composed of collagen and ground substance creating a smooth surface for the epithelium and preventing the penetration of polymorphonuclears and the entry of bacteria, toxins, and chemicals; the stroma, which occupies 90% of corneal thickness and is formed by laminae of collagen fibrils, cells, and ground substance; Descemet's membrane, which is the basement membrane of the endothelium and is formed by very thin collagen fibrils with a uniform distribution; and the endothelium, which is squamous, with an important role in the metabolism of the cornea and in maintaining its transparency [15,16].

The cornea is therefore a natural barrier that prevents pathogens from further invading its stroma. Furthermore, the expression of antimicrobial peptides (AMPs), such as human β-defensin (HBD) and LL-37 (cathelicidin protein) by the epithelium, also contributes to this [17,18].

Moreover, the constant flow of tears can naturally clean the corneal surface from microorganisms and other substances. In addition, tears also contain various molecules with antibacterial properties, such as β-defensins and antimicrobial peptides [19].

The edges of the cornea have a rich distribution of capillaries and lymphatic vessels that serve as entry and exit gates for immune system cells such as dendritic cells (DCs), mast cells (MCs), macrophages, natural killer (NK) cells, γδ T cells and innate lymphoid cells (ILCs) [14].

The increased level of AMPs during fungal keratitis results in the immediate killing of microorganisms and the promotion of other antifungal mechanisms such as increased infiltration by neutrophils [17].

The sclera is the largest part of the fibrous coat of the eyeball and is opaque. In front is the cornea, while at the posterior pole, there is a hole through which the optic nerve passes. Vessels and nerves also pass through smaller holes. The sclera consists of three lobes: the outer episclera, the stroma, and the inner layer that contacts the choroid. The lens of the eye is located behind the iris, on the anterior surface of the vitreous, and is part of the refracting system of the eye [11].

The anterior chamber of the eye is defined as the space between the posterior surface of the cornea, the anterior surface of the iris, and peripherally the sclero-corneal zone. In the cavity of the sclero-corneal zone is the drainage system of the aqueous fluid, which has an important role in the maintenance of the shape of the bulb, the nutrition of the crystalline lens, and the maintenance of normal intraocular pressure [20].

The uveal coat consists of three parts: the iris, the ciliary body, and the choroid. It is the middle vascular coat of the eye and is protected externally by the cornea and sclera [21].

The retina is the inner lining of the eyeball and consists of two lobes: the pigment epithelium and the main retina, which in turn consists of three groups of cells, the optic cells (photosensory receptors), the bipolar cells, and the ganglion cells (optic pathway) [22].

The vitreous is a gelatinous substance located behind the lens and in contact with the retina. Finally, the eyeball is located in the bony cavity of the skull [11].

Filamentous fungal keratitis

Predisposing Factors

All conditions that cause damage to the ocular epithelial barrier favor the entry of fungi into the cornea and the development of inflammation. As mentioned above, trauma by plant material is the most frequent risk factor. The disease mainly affects young male farmers living in developing countries where, according to studies, the rate reaches 24%-83% [7]. On the contrary, the use of contact lenses is the main predisposing factor in developed countries, where the rate reaches 37%-67% [7]. The risk is related to the type of lenses, how often they are replaced, and their cleaning method [7]. The 2005-2006 global outbreak of *Fusarium* keratitis was caused by a particular contact lens cleaning solution [23].

Ocular diseases, such as dry eye, blepharitis, chronic epithelial changes, or inflammations, attack the corneal epithelium and allow fungal penetration into the cornea. Systemic diseases characterized by a reduced immune response such as HIV infection and diabetes mellitus, where in addition, the increased value of glucose in the blood alters the ocular surface and favors the easy attachment and proliferation of fungi, are also significant predisposing factors. Systemic or topical corticosteroid use is another risk factor that affects the immune system and also favors the deeper penetration of the fungus into the cornea leading to a worse outcome. Moreover, a previous ophthalmological operation, such as cataract, laser, or corneal transplantation, also predisposes to the development of fungal keratitis [7].

Immune Mechanisms

The outcome of the disease depends on the virulence of the pathogenic fungus and the host's defense mechanisms. Innate immunity plays an important role in eye protection. The cornea acts as a barrier that protects the intraocular tissue from potential damage. Epithelial cells, which are closely connected, are the first line of defense. In addition, on the ocular surface, there are a large number of substances with anti-inflammatory and bactericidal effects, such as secretory IgA, mucin glycoproteins, and antimicrobial peptides, such as human β-defensin 2 (HBD-2) and LL-37 (cathelicidin), which have been found to be particularly elevated in filamentous fungal keratitis [24,25]. Wang et al. recently suggested that these antimicrobial peptides may not be equally expressed in fungal keratitis since they found HBD-2 mRNAs to be more elevated in *Fusarium* keratitis while LL-37 mRNAs in cases of *Aspergillus* keratitis [17].

Human β-defensin 2 (HBD-2) and LL-37 (cathelicidin) are components of the innate immune system. According to the disulfide bridging array, there are three subgroups of defensins (α-, β-, and θ-defensins). Both α- and β-defensins (HBDs) are key components in local immunity. The α-defensins 1-4 are mainly secreted by neutrophils into tears. HBD-1-3 have been found in the corneal and conjunctival epithelium and the HBD-2 and HBD-3 in the tear film [26]. HBD-1 has also been found in the lacrimal gland and intraocular tissues, while HBD-2 and HBD-3 are commonly produced following invasion by pathogens and inflammatory agents [27,28].

The LL-37 peptide consists of 37 amino acids and is named after the two leucine residues present at the N-terminus of the mature peptide. It has antibacterial, antifungal, antiparasitic, antiviral, and healing action, properties against not only the creation of bacterial biofilms, angiogenesis, and the regulation of apoptosis but also carcinogenesis and metastasis. It is expressed in several epithelial tissues including in conjunctival and corneal epithelial cells [29]. Hou et al. demonstrated that cathelicidin (LL-37) promoted the phagocytosis of conidia by neutrophils and improved the outcome of *Aspergillus fumigatus* keratitis. They also showed that the CXC chemokine receptor 2 (CXCR2) of LL-37 on neutrophils (LL-37/CXCR2) activated phospholipase C-γ2 (PLCγ2), further promoting the process of neutrophil phagocytosis and subsequent autophagy to destroy intracellular conidia [30]. Moreover, Luo et al. showed that LL-37 was able to inhibit the growth and adhesion of *Aspergillus fumigatus* hyphae and the subsequent activation of macrophages and the inflammatory process [31].

Host defense cells, such as macrophages, dendritic cells, and neutrophils, have receptors (pattern recognition receptors {PRRs}) that recognize specific molecules in the cell wall of the pathogenic fungus (pathogen-associated molecular pattern {PAMPs}), such as chitin, β-glucan, and mannan. These receptors (PRRs) include C-type lectin receptors (CLRs), toll-like receptors (TLRs), nucleotide-binding oligomerization domain-like receptors (NLRs), and scavenger receptors (SRs).

CLRs belong to a superfamily of proteins able to recognize carbohydrates in a Ca^2+^-dependent manner. The β-glucan of the fungal cell wall is specifically recognized by dectin-1 (dendritic cell-associated C-type lectin-1) while α-mannan by dectin-2. The recognition by the dectin-1 is followed by the recruitment of neutrophils and macrophages through a process dependent on interleukin (IL) 1β, IL-6, CC motif chemokine ligand 2 (CCL2), CXC motif chemokine ligand 1 (CXCL1), and CXCL2 [32]. Moreover, triggering receptors expressed on myeloid cells-1 (TREM-1) have a synergistic effect with dectin-1 and further enhance inflammation [33]. In addition, the protein caspase recruitment domain-containing protein 9 (CARD9), which is an intracellular protein expressed in cells of the myeloid lineage, such as neutrophils, macrophages, and dendritic cells, is responsible for PRRs signaling to activate innate immunity; the production of inflammatory cytokines and chemokines, such as IL-1β, IL-6, IL-17, interferon-γ (IFN-γ), tumor necrosis factor-α (TNF-α), CXCL1, keratinocyte chemoattractant (KC), CXCL2, macrophage inflammatory protein-2 (MIP-2), and CXCL5; and migration and infiltration by myeloid cells [34,35].

IL-1β and IL-6 are mainly produced by macrophages and lymphocytes. In fungal keratitis, IL-17 is produced by neutrophils, and Th17 cells promote cytokine expression, the initiation of inflammation, and further neutrophil infiltration, causing both fungal death and tissue damage [36]. IFN-γ is mainly produced by T cells and natural killer (NK) cells and stimulates neutrophils, monocytes, and macrophages. TNF-α is mainly produced by macrophages and activates phagocytes. Chemokines CXCL1, CXCL2, and CXCL5 induce neutrophil recruitment [37].

Galectin-3 has recently been reported as a lectin that can activate chitin-associated pattern recognition receptors and regulate neutrophil infiltration and cytokine expression associated with the initiation of adaptive immunity against fungi [38,39].

The macrophage-inducible C-type lectin (Mincle) is expressed in myeloid cells and neutrophils and mostly in macrophages, dendritic cells, and B cells. Mincle receptors participate in innate immunity against fungi by enhancing inflammation [40,41]. Zhao et al. showed that the expression of Mincle was significantly increased at four, eight, 16, and 24 hours after *Aspergillus fumigatus* infection, and this expression was associated with the production of TNF-α, IL-1β, IL-10, and CCL3 in the cornea [41]. Yu et al. showed that in addition to enhancing inflammation, Mincle regulated the formation of nitric oxide (NO), which could cause tissue damage in the eye at concentrations above three times the normal [40]. Lin et al. showed that in keratitis from *Aspergillus fumigatus*, Mincle receptors were able to inhibit neutrophil and macrophage apoptosis through caspase-3 inactivation [42].

Another C-type lectin receptor, surfactant protein D (SP-D), interacts with TLR4, following its stimulation by fungal hyphae. Thus, in cases of keratitis, the SP-D receptor appears to play an immunosuppressive role through the TLR4 signaling pathway [43].

Toll-like receptors (TLRs) are transmembrane receptors. After the recognition of PAMPs, the TLRs activate nuclear factor kappa B (NF-κB) either through the myeloid differentiation factor 88 (MyD88)-dependent or MyD88-independent pathway, triggering the expression of TNF-α, IL-1β, IL-6, IL-8, INF-γ, IL-12, IL-18, and MIP-2; initiating the immune response; and recruiting immune cells, such as macrophages and neutrophils to site of infection [44-46]. Among them, TLR2 and TLR4 are found on corneal epithelial cells and have been shown to play a key role in filamentous fungal keratitis. As previously demonstrated, the TLR2/4-NF-kB signaling pathway is likely essential for the regulation of cytokine and chemokine expression, polymorphonuclear recruitment, and the progression of inflammation [47].

There are also the cytoplasmic receptor nucleotide-binding oligomerization domain-like receptors (NLRs) such as nucleotide-binding oligomerization domain-containing protein-1 (NOD-1), which is increased in filamentous fungal keratitis, and NOD-2, which also promotes the production of pro-inflammatory cytokines, playing an important role in the host immunity. Moreover, there is NOD-like receptor protein 3 (NLRP3), which, in combination with caspase-1, regulates IL-1β expression. Xu et al. demonstrated that calcitonin gene-related peptide (CGRP) can inhibit NLRP3 pathway activation and reduce excessive inflammatory processes in *Aspergillus fumigatus* keratitis [48].

In addition, there are the scavenger receptors (SRs) such as lectin-like oxidized low-density lipoprotein receptor 1 (LOX-1), a membrane receptor mostly expressed on endothelial cells, macrophages, neutrophils, vascular smooth muscle cells, and platelets and which during filamentous fungal keratitis activates inflammation possibly through a mechanism of interaction with TLR4 [49-51]. Scavenger receptors are involved in lipid metabolism and have been found to bind and internalize microorganisms that have lipopolysaccharide (Gram-negative bacteria) or lipoteichoic acid (Gram-positive bacteria) [50]. Li et al. showed that the expression of CXCL1 and TNF-α in human corneal epithelial cells (HCECs) was increased in *A. fumigatus* keratitis via LOX-1. Moreover, *A. fumigatus* rat keratitis activated p38 mitogen-activated protein kinase (MAPK) and increased the expression of CXCL1, TNF-α, and IL-6 again through LOX-1 [50]. Gao et al. demonstrated that LOX-1, TLR4 expression, and reactive oxygen species (ROS) production were increased after *A. fumigatus* infection. Furthermore, they showed that there may have been an interaction between LOX-1 and TLR4 that may then affect the generation of ROS [49].

The scavenger receptor expressed by endothelial cell-Ⅰ (SREC-Ⅰ) is expressed by several cells, such as endothelial cells, epithelial cells, dendritic cells, and macrophages. Zhang et al. showed that SREC-Ⅰ was also expressed in human and murine corneal epithelial cells and that in *A. fumigatus* keratitis, SREC-Ⅰ expression was found to be increased. They also showed that in the case of SREC-Ⅰ inhibition, the production of pro-inflammatory factors such as LOX-1, CXCL1, TNF-α, and IL-1β was attenuated and that the progression of keratitis was slowed [51].

Neutrophils play a key role in immunity against fungal infections. They fight fungi by phagocytosing them, killing them by releasing their granule contents, producing reactive oxygen species (ROS), and forming neutrophil extracellular traps (NETs) [52]. Phagocytosis is mediated by the local polymerization of actin surrounding the pathogen with subsequent endocytosis. In addition to neutrophils, other phagocytes such as dendritic cells, monocytes, macrophages, and also fibroblasts and epithelial and endothelial cells participate. As previously described, the human corneal epithelial cells were able to phagocytose *Aspergillus flavus* conidia, around which an F-actin ring had formed, turning them into phagolysosomes [46].

NETs are fibrous formations of nucleic acids and granular proteins. During the invasion of microorganisms, neutrophils are able to release NETs outside the cell, trap the pathogens, and kill them through toxic proteins. Calprotectin, which is also present in NETs, has been found to bind extracellular zinc from *Aspergillus fumigatus*, inhibiting hyphal growth [53,54]. NETs are released in two main ways. During the first process, the activated peptidylarginine deiminase 4 (PAD4) catalyzes the citrullination of arginine residues resulting in the decomposition of the nuclear envelope and the decondensation of chromatin, which then, together with neutrophil cytoplasmic enzymes such as elastase, cathepsin G, myeloperoxidase, lactoferrin, and gelatinase, form NETs. After the plasma membrane rupture, the NETs are released, while the neutrophils die (NETosis) [55]. During the second process, the NETs are formed through the release of mitochondrial DNA without the ultimate death of neutrophils. In this case, live neutrophils can synthesize NETs containing mitochondrial DNA after the stimulation with granulocyte/macrophage colony-stimulating factor (GM-CSF) and subsequent toll-like receptor 4 (TLR4) or complement receptor 5a (C5a) activation [56,57].

The neutrophils produce nicotinamide adenine dinucleotide phosphate (NADPH) oxidase (NOX), which is responsible for the transformation of molecular O_2_ into superoxide anion (O_2_-) with the simultaneous extracellular release of ROS and protons. Since neutrophils cannot phagocytose hyphae, their ability to inhibit their growth mainly depends on the production of reactive oxidants (ROS), iron sequestration, the limited availability of zinc, and nutrient deprivation [58,59]. Therefore, hyphal survival depends on the interaction between host oxidants and hyphal antioxidants [60]. Leal et al. showed that hyphae activated NOX production via cluster of differentiation (CD) 18, thus causing their death [60]. de Jesus Carrion et al. showed that acidic mammalian chitinase (AMCase), which is mainly expressed in airway macrophages of asthmatic patients, may also be expressed in neutrophils and that neutrophil-derived AMCase together with chitin synthases may play an important role in inhibiting the hyphal growth during *Aspergillus fumigatus* keratitis [59].

However, the excessive production of inflammatory factors can lead to the opposite effects with the destruction of the corneal tissue, ulcer, and worse outcome [61]. The production of ROS can be an important weapon in the fight against microorganisms and the initiation of inflammation, but on the other hand, it can cause damage to mitochondria and tissues. In addition, IL-1β induces ROS production causing the death of hyphae, and vice versa, ROS promotes further production of IL-1β, thereby also destroying the tissues [49]. It has been previously demonstrated that pathogens such as *C. albicans* and *Aspergillus* spp. are able to regulate excessive inflammation by activating the autophagy process that reduces neutrophil recruitment and destroys intracellular microorganisms [62].

Into the stroma, the corneal cells stimulated by fungal hyphae can activate CD4+ T cells, CD8+ T cells, and B cells; promote the production of T-helper (Th) type 2 cytokines (IL-4 and IL-13) and IgG; and increase the proliferation of peripheral blood mononuclear cells (PBMCs) [63,64]. Both the growth of hyphae and the infiltration of neutrophils into the stroma contribute to the loss of corneal transparency and reduced vision. The secretion of proteases, such as matrix metalloproteinase-8 (MMP8)/collagenase by neutrophil granules, causes the destruction of the collagen of the corneal stromal matrix and tissue damage. At this stage of infection, neutrophils have been found to be the predominant source of IL-1β, which is often found elevated during *Fusarium* and *Aspergillus* keratitis [65].

Corneal hypoxia probably plays an important role in the development of fungal keratitis by gradually affecting almost all its cell layers. Lightfoot et al. demonstrated that the first signs of stromal hypoxia in mice with fungal keratitis by *Aspergillus fumigatus* appeared 48 hours after infection due to leukocyte infiltration and subsequent endothelial dysfunction. They also observed that during the progression of the infection, as the corneal edema progressed, the degree of hypoxia increased, and the endothelium decreased. Later, in the following stages, the accumulation of fungal metabolites together with leukocyte ROS/cytokines caused further loss of endothelial integrity and subsequent entry of fluid from the anterior chamber [66].

Additionally, other cellular products also regulate the immune process, such as vasoactive intestinal peptide (VIP), a neuropeptide produced by immune cells with properties regulating the expression of pro- or anti-inflammatory factors and destroying the membrane of microorganisms; maresin 1 (MaR1), a product of docosahexaenoic acid (DHA) expressed on macrophages, able to regulate inflammation by reducing neutrophil recruitment and pathogen burden; and indoleamine 2,3-dioxygenase (IDO) expressed on macrophages, polymorphonuclears, dendritic cells, and epithelial cells, which balances the production of pro-inflammatory cytokines [25].

Huang et al. demonstrated the important role of N6-methyladenosine (m6A)-mediated modification of methyltransferase-like 3 (METTL3) in the development of *Fusarium* keratitis. In their study, they showed that the downregulation of METTL3 slowed the progression of inflammation through the phosphatidylinositol-3-kinase/protein kinase B (PI3K/AKT) signaling pathway and reduced the production of TNF-α, IL-1β, and IL-6, thereby protecting corneal stromal cells [67].

Shi et al. [68] showed that during adaptive immunity in cases of *Aspergillus fumigatus* keratitis in mice, CD3ε activated T-lymphocytes, thus regulating the secretion of IL-10, a cytokine with anti-inflammatory properties. They also revealed that LOX-1 and dectin-1 did not play a role in the regulation of CD3ε expression, which was in contrast to an earlier study by Che et al., in which LOX-1 and dectin-1 regulated IL-10 production in murine *Aspergillus fumigatus* keratitis [69]. Their study concluded that the correct treatment of fungal keratitis was based on maintaining a balance between innate and adaptive immunity [68].

Another study showed that corneal Wnt5a expression was increased in *A. fumigatus* keratitis in patients and mice. Moreover, it was found that dectin-1 and LOX-1 expression, which was dependent on extracellular signal-regulated kinase 1/2 (ERK1/2) and c-Jun N-terminal kinase (JNK) pathways, contributed to this Wnt5a production. This study demonstrated that Wnt5a contributed significantly to the response against fungal pathogens by promoting inflammatory processes such as neutrophil recruitment and cytokine production [70].

Thymic stromal lymphopoietin (TSLP), a cytokine of innate and adaptive immunity that promotes the activation of dendritic cells and contributes to the proliferation and differentiation of T and B lymphocytes, has recently been studied. Moreover, TSLP has been shown to be an important immune factor of corneal epithelium and stroma infected with *Aspergillus fumigatus*. It has also been shown that TSLP could also interact with innate immunity expressed by TLRs in human corneal cells infected with *Aspergillus fumigatus* [64]. TSLP mainly promotes the expression of TLR2, TLR4, and antimicrobial peptides [71].

Han et al. showed that the cyclic guanosine monophosphate (GMP)-AMP synthase (cGAS)-stimulator of interferon genes (STING) signaling pathway activated in human corneal epithelial cells (HCECs) and mouse corneas after *Aspergillus fumigatus* infection played an important role in promoting inflammation, mainly through the production of cytokines such as TNF-α, IL-1β, IL-6, and IFN-β, and in disease progression [72].

Despite the progress in research on the immune mechanisms of filamentous fungal keratitis, many fields are still unexplored. It is a multifactorial disease with many challenges that additionally requires both in vitro and in vivo studies for correct conclusions in order to understand and properly treat it.

Fungal species involved in filamentous fungal keratitis

*Fusarium* is the most common cause of keratitis worldwide [73]. They are widely distributed in the soil, subsoil, and plants, while they have additionally been detected in the air and the aquatic environment of the sea [74].

In humans, they cause a wide range of infections, superficial (onychomycosis and keratitis) in immunocompetent and deep in immunocompromised [74]. Deep infections are most commonly caused in patients with neutropenia and/or T-cell immunodeficiency, hematological malignancies, and more recently COVID-19 [75].

*Fusarium* keratitis is considered an emergency. Without proper treatment, the inflammation progresses inexorably to perforation, endophthalmitis, and eventually loss of the eye. It is estimated that 42%-52.5% of all cases of fungal keratitis are caused by *Fusarium* species with the majority occurring in tropical countries, involving young male farmers, following injury. In temperate countries, cases of *Fusarium* keratitis are more common in females and are associated with contact lens wear. *Fusarium* keratitis outcome depends on strain characteristics and host immune response [73]. The temperature inside the cornea (32.6±0.700°C) is ideal for the growth of this fungus [74]. Sometimes, it invades the anterior chamber where it forms a mass in the pupil area, preventing the normal drainage of the aqueous humor and resulting in increased intraocular pressure [76,77].

However, biofilm formation is the main virulence factor, also contributing to the resistance of the strains to antifungals [73]. The ability of *Fusarium* to form biofilms has been studied in vitro in contact lens models, and in vivo in diseased corneas. Proteins involved in biofilm formation can promote angiogenesis, adhesion, infiltration, and immunomodulation [73]. *Fusarium* also secretes enzymes such as carboxypeptidases, aminopeptidases, and mycotoxins that make it highly toxic and capable of causing corneal ulcers. It promotes the activation of macrophages, the infiltration of polymorphonuclear cells, and the increase in the levels of cytokines such as IL-1β, IL-8, IL-17, and TNF-α. The immune response is mediated by receptors such as toll-like 2 and 4 and vitamin D receptors. In addition, it causes changes in the protein profile of the host's tears [73].

*Aspergillus* is the second most common cause of keratitis worldwide, and most cases also occur in tropical and subtropical countries. *Aspergillus fumigatus*, *A. flavus*, *A. niger*, and *A. terreus* are most commonly involved [8,9]. A major risk factor is the trauma by plant material, through which the conidia penetrate the corneal epithelium and invade the stroma. There, the conidia form hyphae, which can migrate through the stroma to the anterior chamber and posterior eye. The hyphae activate local macrophages to produce chemokines that mediate neutrophil recruitment from the capillaries, resulting in corneal opacification, decreased vision, and, in severe cases, vision loss [59].

*Aspergillus* conidia are coated with a layer of hydrophobic proteins, the RodA hydrophobins, which cover the cell wall molecules responsible for initiating the immune response [78]. In the absence of RodA (RodA mutants), these molecules, mainly β-1,3-glucan and α-mannan, remain exposed in the cell wall of the conidia and can be recognized by the receptors of host defense cells, mainly dectin-1 and dectin-2, which then are activated, causing neutrophils to migrate into the cornea to fight the fungus. On the contrary, when RodA proteins are present, they coat the β-1,3-D-glucan and α-mannan of the conidia surface, blocking their recognition by the dectin-1 and dectin-2 lectins, thereby preventing macrophage cytokine production and infiltration by neutrophils and enhancing the fungal survival in the cornea [78,79].

Melanin in *Aspergillus* species has also been found to inhibit Ca^2+^/calmodulin-induced light chain 3 (LC3)-associated phagocytosis (LAP), which is an alternative autophagy pathway [80]. In addition, melanin contributes to the stability and integrity of the cell wall and to the enhancement of virulence in *Aspergillus fumigatus* and *Aspergillus flavus* by protecting them from the host defense. Rudhra et al. demonstrated in an in vivo virulence study that conidia of *A. flavus* strains covered by a melanin layer were more virulent than uncoated ones [81].

Keratitis caused by filamentous fungi, such as *Fusarium* and *Aspergillus*, if not treated properly and promptly, gradually worsens into endophthalmitis. In these cases, the hyphae spread throughout the layer and create a feathered appearance in the shape of the infiltrate and satellite lesions. These cases, in a large percentage, after a period of failed treatments, result in a corneal transplant [82].

Phaeohyphomycetes, mainly *Curvularia* species (formerly genus *Bipolaris*), are the third most common cause of keratitis worldwide. Other species involved are *Exserohilum* spp., *Alternaria* spp., *Ulocladium* spp., *Lasiodiplodia* spp., and *Colletotrichum* spp. [7]. The immune mechanisms and the predisposing factors are similar to other filamentous fungi. However, in keratitis by Phaeohyphomycetes, the cornea exhibits characteristic pigmented plaques that prevent the penetration of topical antifungals, often resulting in superficial keratectomy [83].

*Alternaria* species are found everywhere in the soil, on plants, and in food. They can cause opportunistic infections in humans, including skin, subcutaneous, and ocular infections; rhinitis; and onychomycosis [84]. Traumatism, previous eye surgery, and preexisting corneal diseases are most commonly associated with *Alternaria* keratitis. However, *Alternaria* keratitis cases associated with soft contact lens wear have also been reported [85-87].

*Phoma* species are considered phytopathogenic and are widely distributed in the environment, especially in aquatic systems and soil. In a previous study, 32 *Phoma* infections were reported. The majority was caused by traumatism (22/32, 69%), and 5/32 (16%) were ocular infections [88]. The treatment usually requires the surgical excision of the infected tissues and the administration of antifungal treatment locally and/or systemically.

*Curvularia* spp. (former genus *Bipolaris*) have a worldwide distribution. They are mostly phytopathogenic, but they are able to cause infections in both immunocompetent and immunocompromised patients, such as sinusitis, keratitis, endophthalmitis, onychomycosis, dialysis-related peritonitis, and pulmonary and skin infections, mainly in tropical and subtropical areas [89]. *Curvularia spicifera* and *C. hawaiiensis* are the most frequent causes of keratitis [90].

*Purpureocillium lilacinum* (formerly *Paecilomyces lilacinus*) belongs to ascomycetes from the Ophiocordycipitaceae family. They are found in soil, decaying material, insects, and sea jellyfish [91]. The risk factors of keratitis include chronic ocular disease, a previous eye surgery, corneal trauma, or contact lens wear [92]. Cases with other predisposing factors such as immunosuppression, the use of systemic immunosuppressants, and topical corticosteroids have also been described [93]. Furthermore, serious ocular infections in immunocompetent individuals have recently been reported [94]. Infections associated with sterile sodium bicarbonate solution, skin lotions, and solutions used to sterilize artificial lenses have also been recorded [95,96].

*Beauveria bassiana* is an entomopathogenic fungus. This species rarely infects humans [97]. Only four cases of opportunistic infections in the immunocompromised and 15 cases of keratitis in contact lens wearers or after ocular trauma have been documented [98-100]. It grows adequately at temperatures of 35°C-37°C, and perhaps, this explains why the infections are limited to superficial body tissues such as the cornea [98].

*Acremonium* is an environmental saprophyte that has been isolated from soil and plant debris, which causes opportunistic superficial infections in humans, respiratory tract infections, onychomycosis, and ocular fungal infections. The main risk factor for ocular infections is injury with contaminated plant material, followed by the previous use of steroids or broad-spectrum antibiotics, poor eye condition, strabismus, and contact lens wear [101]. *Acremonium* has also been implicated in causing keratitis after the laser-assisted in situ keratomileusis (LASIK) procedure and has been detected in the operating room environment. The host's immune system probably also plays a key role. Psoriasis, Hansen's disease, lagophthalmos, diabetes, tuberculosis, and also Sjögren's syndrome under systemic immunosuppression have been associated with *Acremonium* keratitis [101,102].

*Penicillium* fungal keratitis usually affects during winter and monsoon and more often young male farmers with a history of trauma or under some type of immunosuppression. Furthermore, *Penicillium marneffei* keratitis has been found to be associated with AIDS [103].

*Scedosporium* spp. are causes of life-threatening infections in immunocompromised patients. They are also responsible for causing eumycetoma, a chronic deep fungal infection of the skin and subcutaneous tissues. They also affect many organs in the body, including the bones and joints, the central nervous system, the upper and lower respiratory system, and the eyes. Several predisposing factors have been mentioned such as cystic fibrosis, hematopoietic stem cell and solid organ transplantation, and COVID-19 [104]. Injury mainly by plant matter, uncontrolled diabetes, and small-incision cataract surgery have also been reported as risk factors for *Scedosporium* keratitis [105].

## Conclusions

The immune response in filamentous fungal keratitis is initiated by the recognition of fungal PAMPs and the subsequent activation of corneal PRRs and involves the vasodilatation and secretion of active immune cells, such as macrophages, polymorphonuclear leukocytes and lymphocytes, and immunoreactive substances. However, the overexpression of inflammatory cytokines and chemokines could lead to adverse effects even in corneal ulceration or perforation. Therefore, filamentous fungal keratitis is a particularly serious infection that can lead to reduced vision and even blindness not only because of the invasiveness and difficulty of treating these microorganisms but also because of the excessive inflammatory host response. It is possible that antifungal therapy in combination with topical immunosuppressants may be the most effective strategy to improve the clinical outcome of the disease. However, more research is needed for safer conclusions.
